# Probabilistic 3D Reconstruction Using Two Sonar Devices

**DOI:** 10.3390/s22062094

**Published:** 2022-03-08

**Authors:** Hangil Joe, Jason Kim, Son-Cheol Yu

**Affiliations:** 1 Department of Robot and Smart System Engineering, Kyungpook National University, 80 Daehak-ro, Buk-gu, Daegu 41566, Korea; hgjoe@knu.ac.kr; 2 Department of Convergence IT Engineering, Pohang University of Science and Technology, 77 Cheongam-ro, Nam-gu, Pohang 37673, Korea; js21kim@postech.ac.kr

**Keywords:** sonar data processing, 3D reconstruction, sensor fusion, forward-looking sonar, profiling sonar, underwater sensing, acoustic images, sonars

## Abstract

Three-dimensional reconstruction is a crucial technique for mapping and object-search tasks, but it is challenging in sonar imaging because of the nature of acoustics. In underwater sensing, many advanced studies have introduced approaches that have included feature-based methods and multiple imaging at different locations. However, most existing methods are prone to environmental conditions, and they are not adequate for continuous data acquisition on moving autonomous underwater vehicles (AUVs). This paper proposes a sensor fusion method for 3D reconstruction using acoustic sonar data with two sonar devices that provide complementary features. The forward-looking multibeam sonar (FLS) is an imaging sonar capable of short-range scanning with a high horizontal resolution, and the profiling sonar (PS) is capable of middle-range scanning with high reliability in vertical information. Using both sonars, which have different data acquisition planes and times, we propose a probabilistic sensor fusion method. First, we extract the region of interest from the background and develop a sonar measurement model. Thereafter, we utilize the likelihood field generated by the PS and estimate the elevation ambiguity using importance sampling. We also present the evaluation of our method in a ray-tracing-based sonar simulation environment and the generation of the pointclouds. The experimental results indicate that the proposed method can provide a better accuracy than that of the conventional method. Because of the improved accuracy of the generated pointclouds, this method can be expanded for pointcloud-based mapping and classification methods.

## 1. Introduction

Three-dimensional reconstruction from acoustic images is an important task in underwater sensing because an acoustic sensor, that is, image sonar, is robust to water turbidity. Because of the current improvement in sonar technologies, forward-looking multibeam sonars (FLSs) provide high-resolution 2D acoustic images that are similar to optical images from a camera [1]. However, compared to optical images, acoustic images experience quality degradation caused by the image-generating mechanism, such as the loss of elevation information, perceptual ambiguity, and a low signal-to-noise ratio [2]. These drawbacks complicate 3D reconstruction, including pointcloud generation using FLS.

For 3D reconstruction with sonar images of FLS, additional constraints are required, which categorize the approaches for 3D reconstruction using FLS. One approach is shape from shading [3,4]. The results from this approach depend on the environmental condition of the seabed; thus, precise calibration is required for the environment. Another approach is the use of feature-based methods, which are conventional approaches in computer vision algorithms, such as structure from motion (SfM, [5,6,7]). However, feature-based methods are difficult to implement in practical applications because of noisy data caused by interference, specifically with the background in sonar images, and perceptual ambiguity. Multiple-imaging sonar views of a scene, such as space carving [8,9] and deconvolution [10], are another approach. To obtain multiple images around an object of interest, the autonomous underwater vehicle (AUV) with FLS should remain at a certain site or reroute the trajectories that consume unnecessary time and energy. To prevent unnecessary rerouting, Cho et al. proposed an incremental 3D pointcloud generation method with a forward-moving AUV [11]; however, vertical ambiguity due to the vertical beamwidth caused an improper slope in the front area of the object of interest, thus degrading the quality of the resulting pointcloud. To improve the quality of the generated pointcloud, Joe et al. proposed another method for underwater 3D reconstruction, which uses two sonar devices with complementary information [12]; this method requires a segmentation process for the front slope of an object in the pointcloud, which increases the computational load. In addition, a Monte-Carlo-based approach for 3D reconstruction was introduced in [13], which presented a method using a likelihood map generated by profiling sonar (PS) data and utilized it for reconstructing the elevation information of FLS data. A drawback of this method is biased dependence on PS information, so horizontal accuracy is not guaranteed. To overcome the limitation, we propose an improved method using the probabilistic sensor model and importance sampling with combined weight calculation.

In this paper, we present and address an improved method for 3D reconstruction that is applicable for multiple objects on the seabed by using a probabilistic approach with two sonar devices: FLS and PS. FLS is a high-frequency multibeam sonar capable of short-range scanning with high horizontal resolution, while PS is capable of middle-range scanning with low horizontal resolution but high reliability of vertical scanning. To exploit complementary information, we adopted a crossed installation of two sonar devices in such a way that the PS was laid down on its side and mounted on top of the FLS. From this installation, the FLS scanned reliable horizontal information and the PS scanned the vertical profile of the middle-range front area of the AUV. The fusion method is addressed in Section 2, including the extraction of a region of interest (ROI), probabilistic sensor model, and improved weight calculation. Through the sensor fusion of the complementary characteristics of both sonars, 3D information is reconstructed. The proposed method was verified using simulations and experiments. The experiment was conducted using a hovering-type AUV in a real sea. The proposed method can generate 3D pointclouds of vertically extruded objects deployed side by side, which can be applied for underwater mapping and the search for small objects by using the pointcloud-based classification method [14].

## 2. Method

### 2.1. Characteristics of Sonar Imaging

**Figure 1:**
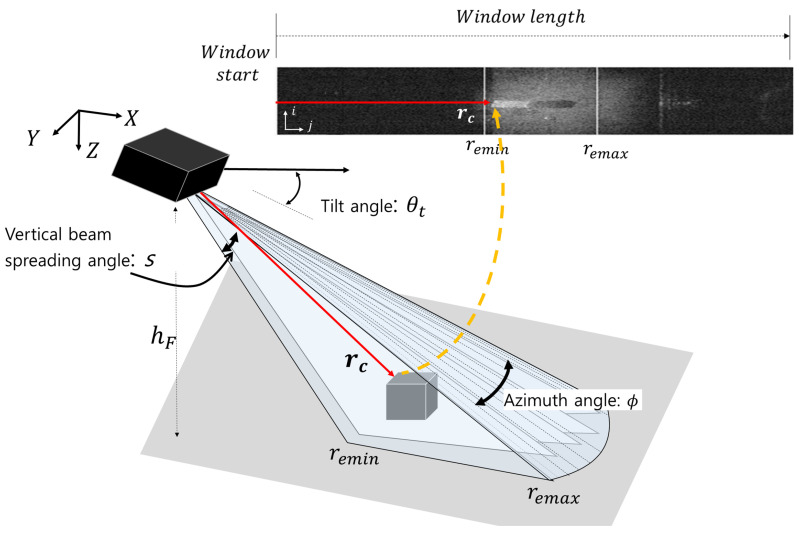
Acoustic beam geometry of the FLS and sonar image generation.

**Figure 2:**
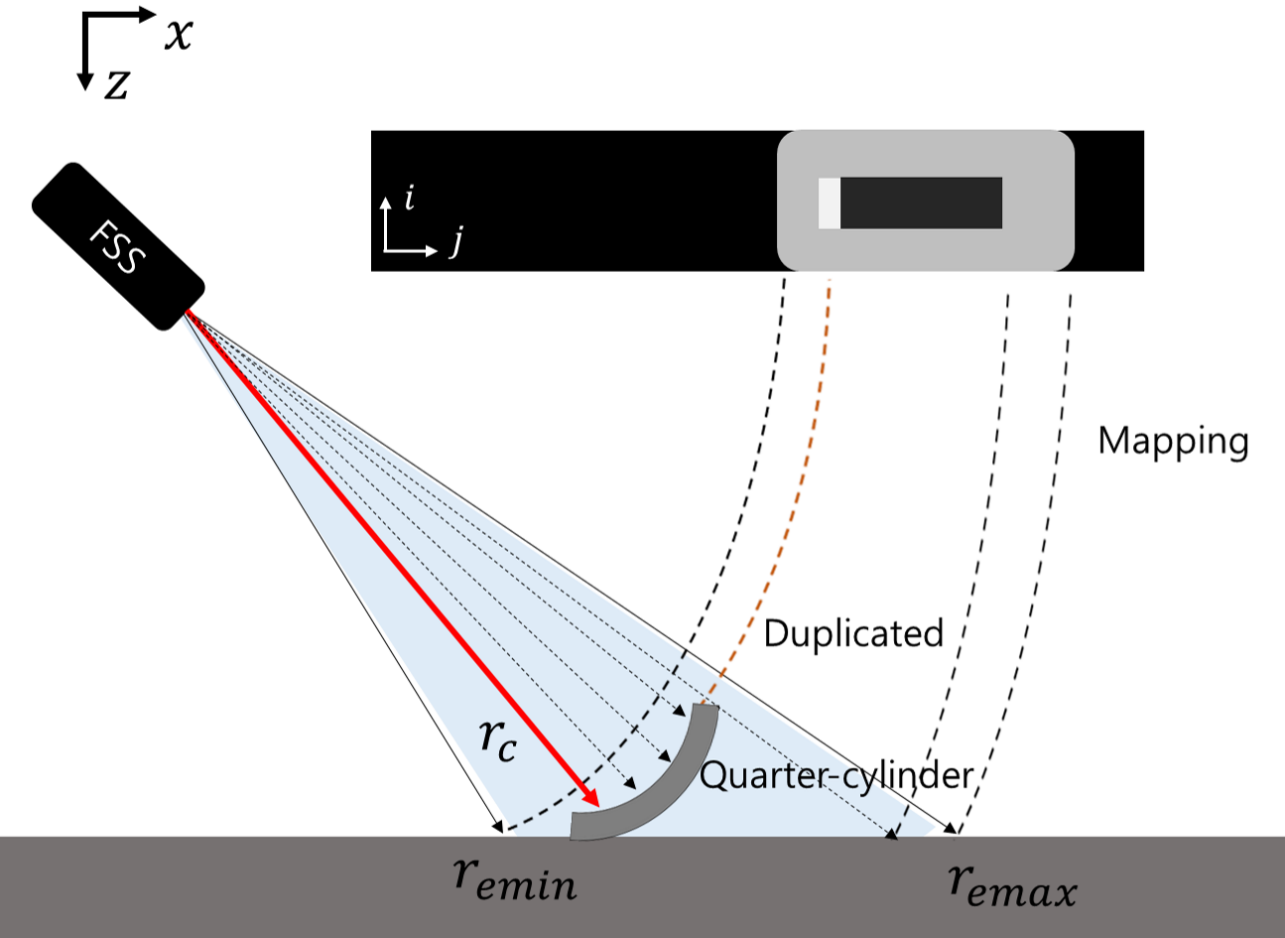
Acoustic beam geometry and image generation of the FLS in a 2D vertical view.

The FLS consisted of 96 transducers with a linear arrangement, and it synthesized fan-shaped beams with 29${}^{\circ }$ and 14${}^{\circ }$ in horizontal and vertical spreading angles, respectively. The returned beams were synthesized into an acoustic image with a size of 512 × 96, as shown in Figure 1. For acoustic beam geometry, let the altitude, tilt angle, azimuth angle, and vertical beam spreading angle of the FLS be $h_{r}$, *t*, ϕ, and *s*, respectively. $r_{c}$ represents the returned beam at the top- and front-most part of an object. The field of view (FOV) of the FLS is determined by $r_{emin}$ and $r_{emax}$, which are related to the vertical spreading angle, tilt angle, and altitude of the FLS. In the FOV, acoustic beams returned at the equal range are mapped into the same point (Figure 2), which causes the loss of elevation information. If the object shapes are complex and protrude irregularly, the loss of information causes perceptual ambiguity. Speckle noise also degrades sonar image quality. Speckle noise is caused by an interference of the coherent return signals, and this granular noise causes a low SNR and blurred effect on the boundary of an object in a sonar image. A noisy background is also one of the difficulties in sonar image processing. The background is a collection of returned beams backscattered from the seafloor, which is a mixture of coarse particles, such as sand and small rocks. Generally, they have a good acoustic reflectivity; thus, the background tends to have high intensity, which hinders target object segmentation. Because of those reasons, conventional computer vision algorithms suffer in sonar image processing.

### 2.2. Limitation in the Single-Sonar Method

Another difficulty in 3D reconstruction with sonar images is the uncertainty in elevation information caused by the beamwidth of acoustic waves. The uncertainty in elevation information increases as the angle of the sonar beam increases. In order to figure out the relation, an additional simulation was conducted. In the simulation, we deployed a single object with different front slopes and generated a pointcloud using Cho’s method in [11]. Six objects with different front slopes were used in the simulation, as presented in Table 1. Sonar models were applied in the same configuration in the formal simulation, but the sonar tilt angle was set to 30${}^{\circ }$. The sonar moved forward while maintaining an altitude of 2.5 m depth.

**Figure 3:**
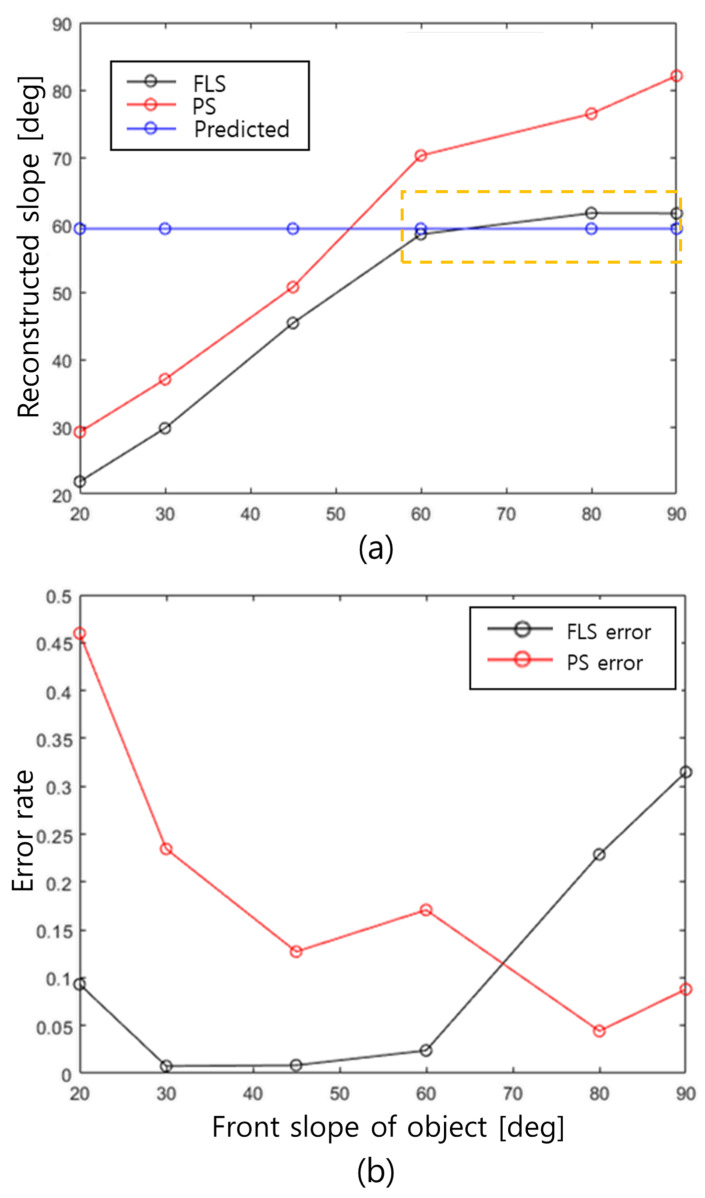
Estimated (**a**) slope and (**b**) error rate with different front slopes of objects; black and red circles denote FLS and PS results, respectively. The blue circles in (**a**) represent the predicted values from Equation (4).

The results are presented in Figure 3. The uncertainty caused by the beamwidth was determined by estimating the front slope of the generated pointcloud, which is shown in Figure 3a. The black and red circles represent the results from the FLS and PS, respectively. Figure 3b shows the errors with respect to the slope of the input object. The error of the PS decreased gradually as the slope of the object increased, while the error of FLS dramatically increased from the case for the object with the slope of 60 degrees. The slope where the error increases is called the slope limit, which can be predicted using geometric analysis.

**Figure 4:**
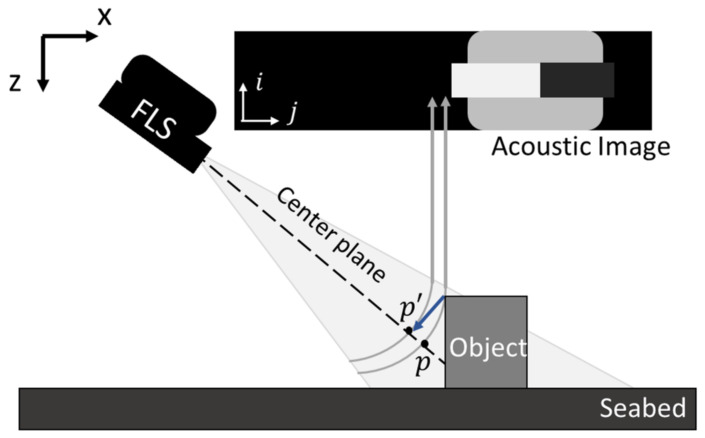
Orthographic projection approximation. *p* is the original point of *P* mapped by sonar geometry, and $p^{\prime }$ is the projected point of *P* on the center plane of the acoustic beam.

The slope limit can be modeled. Given that the elevation angle is sufficiently narrow, the orthographic projection approximation (Figure 4) is valid [15,16], and the difference between points $p^{\prime }$ and *p* in Figure 4 becomes negligible.

**Figure 5:**
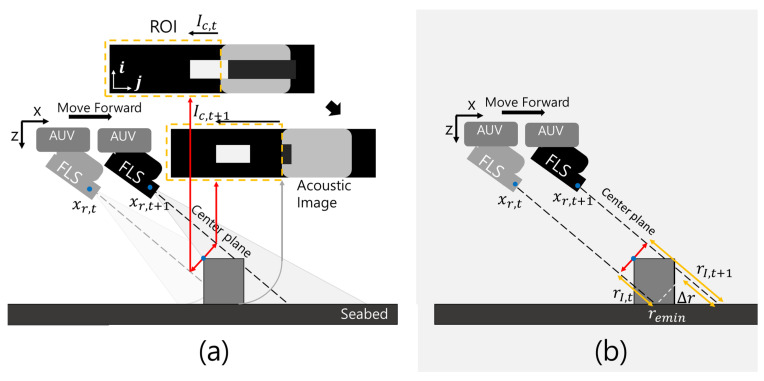
Change in length of the highlight in the front area of the sonar image with the change in sonar position. (**a**) presents highlight changes in sonar images of FLS, and (**b**) shows the corresponding changes in range.

When an AUV equipped with an FLS moves forward, variations in the highlights in the image can be described, as shown in Figure 5. If the AUV maintains its altitude and the tilt angle of FLS remains constant, the position changes of the FLS are coincident with the location changes of the AUV. The position changes are denoted by $x_{r,t}$ to $x_{r,t+1}$, and the highlight length in the image plane increases from $I_{c,t}$ to $I_{c,t+1}$ (Figure 5a), which is based on the sonar image generation mechanism of FLS. Based on the sonar projection geometry, $I_{c,t+1}-I_{c,t}$ is approximated as f(Δr) (Figure 5b), where f(·) is the function for transformation into pixel space. The following relation is derived:

(1) $$\begin{array}{c} \Delta r=\Delta x\cos(\theta_{t}+\theta_{r}), \end{array}$$

where $\theta_{t}$ and $\theta_{r}$ are the tilt of the FLS and the pitch angle of an AUV equipped with an FLS, respectively.

In the pointcloud map *M*, the slope of the points can be derived [12]. Given that the derivatives between the points generated in the X-Z plane are as follows:

(2) $$\begin{array}{cc} \Delta x_{t} & =\Delta x_{r}\\ \; & +\Delta r_{c}\sqrt{1-\sin^{2}\left(t+s/2+\theta_{r}\right)}, \end{array}$$

(3) $$\begin{array}{cc} \Delta z_{t} & =\Delta z_{r}-\Delta r_{c}\left(j\right)\sin\left(t+s/2+\theta_{r}\right), \end{array}$$

where $\Delta r_{c}\approx -\Delta x_{r}\cos\left(\theta_{t}+\theta_{r}\right)$ and $z_{r}$ is constant, then the slope is obtained:

(4) $$\begin{array}{cc} \alpha & =\frac{z_{t}}{x_{t}}=\tan^{-1}\left(\frac{\cos\left(t+\theta_{r}\right)\sin\left(t+s/2+\theta_{r}\right)}{1-\cos\left(t+\theta_{r}\right)\cos\left(t+s/2+\theta_{r}\right)}\right). \end{array}$$

where *s* is a beamwidth of an acoustic wave. Usually, *s* is non-zero, and sonars are installed to look forward and downward on an AUV. Therefore, the front slope always occurs if an object on the seabed has a shape that is vertically extruded and has a relatively small size. Therefore, there is a limitation in methods using single sonar, and we propose a combination of two sonar devices.

### 2.3. Proposed Method

**Figure 6:**
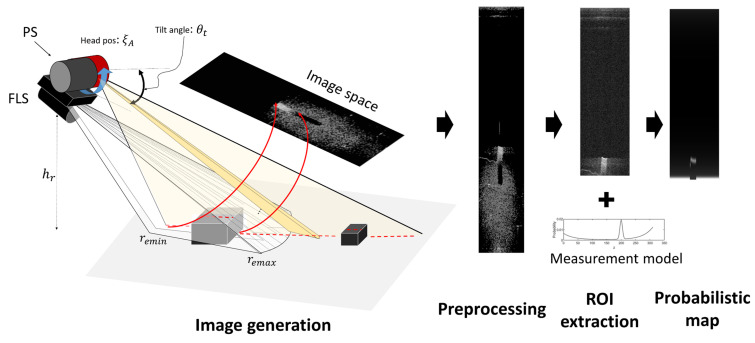
FLS measurement model.

The proposed method of fusing two complementary types of data from two sonar devices consists of two stages: (1) extracting the region of interest (ROI) and (2) probabilistic point extraction (Figure 6). The FLS is installed in a forward-looking orientation, and the axis of the transducer array is horizontal. The other sonar is installed in such a way that the two sonars have a vertically crossed installation. The PS rotates its transducer vertically and acquires forward vertical information. From the installation, the FLS scans horizontal information, and the PS obtains a vertical profile of the front area of the AUV. Because the two sonar devices have different data acquisition planes and times, the obtained data are complementary to each other. The FLS acquires short-range data with reliability in the horizontal direction, whereas the PS acquires far-distance data with reliability in the vertical direction, and it acquires the data earlier than the FLS. Because the two sonars emit fan-shaped beams, their data have high uncertainties in the vertical and horizontal directions, respectively. The proposed method for mitigating uncertainties is divided into two steps. The first is an iterative data acquisition and occupancy-grid-based recursive update, which generate a likelihood field for the vertical information. The second is importance-sampling-based most-likely point extraction, which uses the generated likelihood map. The iterative data acquisition and recursive update are conducted using the PS, and importance-sampling-based 3D pointcloud generation is conducted using the FLS.

### 2.4. Region of Interest

**Figure 7:**
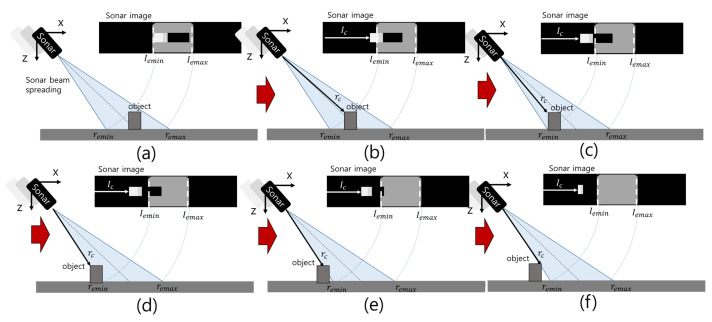
Stepwise changes in the sonar image when the sonar approaches an object from (**a**–**f**).

Scattered reflections from the seabed form a background around the object, which hinders the extraction of objects. To avoid the difficulty of the background, we set the ROI to be different from the FOV. As the FLS on an AUV approaches an object on the seabed, the highlight in the sonar image changes (Figure 7). When the object is located in the FOV, the highlight is in the background. As the FLS approaches the object, the highlight is not in the background. Outside the background, we can extract highlights returned from the object area without disturbances from the background. The width and length of the object can be estimated by analyzing the width and length of the highlight in that area, and even the height information can be obtained by measuring the maximum reach of the highlight outside of the background. This is called the highlight extension effect (HEE) [11]. We set the region outside the background as the ROI in the sonar image to reduce the effect from the background.

The ROI is defined by calculating $r_{emin}$. Let the sonar return data be I(i,j) and let the *i*-th row of I(i,j) be $I_{r}\left(i\right)$, where i={1⋯n} and j={1⋯m}. Given the altitude, $h_{r}$, of the FLS, $r_{emin}$ and the corresponding pixel index $I_{emin}$ in the image space are obtained as follows:

(5) $$\begin{array}{cc} r_{emin} & =\frac{h_{r}}{\sin(\theta_{t}\left(\xi \right)+s/2)},\;\;I_{emin}=\left[n\frac{r_{emin}-r_{min}}{r_{max}-r_{min}}\right], \end{array}$$

where ξ is the azimuth angle, where −14.5 <ξ< 14.5, and [·] is the nearest integer function. *i* is the maximum number of bins, and *j* is the number of the transducer. Here, i×j is 512 × 96, which is the size of the sonar image. $r_{min}$ is the predefined window start, which is illustrated in Figure 2. Considering *j*, each transducer has an ROI, which is denoted by S(j), and it is defined as follows:

(6) $$\begin{array}{c} \mathrm{ROI}\;S\left(j\right):\;\mathrm{set}\;I_{r}\left(i,j\right)\in S\left(i,j\right)\;\mathrm{that}\;I_{r}\left(i,j\right)\leq I_{emin}. \end{array}$$

### 2.5. Sonar Measurement Model

Given the robot pose *x* and map *m*, the FLS measurement model can be considered a probability sensor model. To deal with uncertainty in the elevation angle, the measurement of the FLS is divided into range and elevation angles as follows:

$$\begin{array}{cc} p(z_{F}|x,m)= & \;p(\xi ,r|x,m) \end{array}$$

(7) $$\begin{array}{cc} \overset{\mathrm{Bayes}}{=} & \;\frac{p(\xi |r,x,m)p(r|x,m)p(x|m)p(m)}{p(x|m)p(m)} \end{array}$$

(8) $$\begin{array}{cc} =\; & \eta p(\xi |r,x,m)p(r|x,m), \end{array}$$

where $z_{F}$ is a measurement of the FLS; *r* and ξ are the range measurement and elevation angle, respectively. Assuming that measurements from each transducer are independent and the noise model of the FLS is Gaussian, the distribution of p(r|x,m) can be modeled using the Gaussian mixture. The mixture distribution of p(r|x,m) is presented as

(9) $$\begin{array}{cc} p(r|x,m)= & \alpha_{1}p_{1}\left(r|x,m\right)+\alpha_{2}p_{2}\left(r|x,m\right)+\alpha_{3}p_{3}\left(r|x,m\right)\\ \; & +\alpha_{4}p_{4}\left(r|x,m\right), \end{array}$$

where $p_{1}$ is the measurement noise for estimating the critical point $r_{c}$; $p_{2}$ is the noise from unexpected objects, such as fish; $p_{3}$ is the random noise; $p_{4}$ is the measurement noise for estimating $r_{emin}$, which is caused by an uneven seafloor. $\alpha_{1,2,3,4}$ are parameters to be calibrated to satisfy ∑p(r|x,m)=1. Each is described as

(10) $$\begin{array}{cc} p_{1}\left(r|x,m\right) & =\eta \frac{1}{\sqrt{2\pi \sigma }}\exp\left(-\frac{{\left(r-{\hat{r}}_{c}\right)}^{2}}{2\sigma }\right), \end{array}$$

(11) $$\begin{array}{cc} p_{2}\left(r|x,m\right) & =\left\{\begin{array}{cc} \eta \lambda \exp(-\lambda r) & r<r_{c}\\ 0 & otherwise \end{array}\right. \end{array}$$

(12) $$\begin{array}{cc} p_{3}\left(r|x,m\right) & =\eta \frac{1}{r_{emin}}, \end{array}$$

(13) $$\begin{array}{cc} p_{4}\left(r|x,m\right) & =\eta \lambda \exp(\lambda r). \end{array}$$

where η and λ are normalizers; ${\hat{r}}_{c}$ is the expected critical point; σ is the variance of the noise in the range measurement.

Because the elevation information overlaps at one point, as shown in Figure 2, p(ξ|r,x,m) is unknown. Therefore, we propose the addition of an additional sonar measurement $z_{P}$. Given the additional sonar measurement $z_{P}$, the fused model is presented as

(14) $$\begin{array}{cc} p\left(\xi ,r|z_{P},x,m\right)\overset{\mathrm{Bayes}}{=}\; & \frac{p(\xi |r,z_{P},x,mp\left(r|z_{P},x,m\right)p\left(z_{P},x,m\right)}{p(z_{P},x,m)} \end{array}$$

(15) $$\begin{array}{cc} =\; & \eta p\left(\xi |r,z_{P},x,m\right)p\left(r|z_{P},x,m\right) \end{array}$$

Here, $p(\xi |r,z_{P},x,m)$ is

(16) $$\begin{array}{cc} p\left(\xi |r,z_{P},x,m\right)\overset{\mathrm{Bayes}}{=} & \;\frac{p\left(r|\xi ,z_{P},x,m\right)p\left(\xi |z_{P},x,m\right)p\left(z_{P},x,m\right)}{p\left(r|z_{P},x,m\right)p\left(z_{P},x,m\right)} \end{array}$$

(17) $$\begin{array}{cc} = & \;\eta \frac{p\left(r|\xi ,z_{P},x,m\right)p\left(\xi |z_{P},x,m\right)}{p(r|z_{P},x,m)}. \end{array}$$

Therefore,

(18) $$\begin{array}{c} p\left(\xi ,r|z_{P},x,m\right)∼p\left(r|\xi ,z_{P},x,m\right)p\left(\xi |z_{P},x,m\right). \end{array}$$

If $p(\xi |z_{P},x,m)$ can be obtained by sampling from the additional sonar measurement, this assumption allows us to estimate the most likely hypothesis of the elevation angle.

### 2.6. Likelihood Field Generation

Given that the PS scans the same area as the FLS, we can approximate $p(\xi |z_{P},x,m)$ as a Gaussian distribution. Using this approximation, the most likely measurement of the FLS in Equation (18) is obtained using importance sampling. Let the proposal distribution be $p(\xi |z_{P},x,m)$; then, the individual importance weight $w_{t}^{\left(i\right)}$ is assigned to each hypothesis of the FLS measurement as

(19) $$\begin{array}{cc} w_{t}^{\left(i\right)}= & \frac{p(\xi ,r|z_{P},x,m)}{p(\xi |z_{P},x,m)}∼p\left(r|\xi ,z_{P},x,m\right) \end{array}$$

(20) $$\begin{array}{cc} = & \int p\left(r|\xi ,z_{P},x,m^{\prime }\right)p\left(m^{\prime }|z_{P},x\right)dm^{\prime }, \end{array}$$

where $m^{\prime }$ is the likelihood map generated by the PS.

$p(m^{\prime }|z_{P},x)$ is the inverse sensor model, which gives us the occupancy probability. Because the PS obtains range measurements by rotating its transducer head to the preset head positions, the probability is obtained by counting the reflected beams at every cell. Unlike the FLS, the PS obtains range measurements by rotating its single transducer and synthesizes them using a known head position. Therefore, a bundle of scans of the PS is effective, and it is used for sensor fusion. The bundle of scans of the PS is represented as $p(m^{\prime }|z_{P,t-k:t},x_{t-k:t})$, which is obtained using the occupancy grid scheme with the following logarithmic representation:

(21) $$\begin{array}{cc} l(m^{\prime }|z_{P,t-k:t},x_{t-k:t}) & =l(m^{\prime }|z_{P,t},x_{t})\\ \; & +l(m^{\prime }|z_{P,t-k-1:t-1},x_{t-k-1:t-1})\\ \; & -l(m^{\prime }), \end{array}$$

where $p(m^{\prime }|z_{P,t-k:t},x_{t-k:t})$ is calculated with

(22) $$\begin{array}{c} p\left(m^{\prime }|z_{P,t-k:t},x_{t-k:t}\right)=1-\frac{1}{1+\exp l(m^{\prime }|z_{P,t-k:t},x_{t-k:t})}. \end{array}$$

For $p(r|\xi ,z_{P},x,m^{\prime })$, we can simplify it by extracting the range information of the FLS in the sonar image. We can apply the difference filter after Gaussian filtering on the ROI S(i,j) [12] as follows:

(23) $$\begin{array}{cc} S_{G}\left(i,j\right) & =\sum_{k=-2}^{2}S\left(i+k,j\right)G\left(k,j\right), \end{array}$$

(24) $$\begin{array}{cc} S_{D}\left(i,j\right) & =\sum_{k=-1}^{1}S_{G}\left(i+k,j\right)D\left(k,j\right), \end{array}$$

where

(25) $$\begin{array}{cc} \; & G=[{\bar{g}}_{1}\left(x;\sigma \right),\cdots ,{\bar{g}}_{m}\left(x;\sigma \right)], \end{array}$$

(26) $$\begin{array}{cc} \; & g_{i}\left(x;\sigma \right)=\frac{1}{\sqrt{2\pi }\sigma }exp\left(\frac{-x^{2}}{2\sigma^{2}}\right):x={\left[-2,-1,0,1,2\right]}^{T}, \end{array}$$

(27) $$\begin{array}{cc} \; & {\bar{g}}_{i}\left(x;\sigma \right)=g_{i}\left(x;\sigma \right)/\sum_{k=-2}^{2}g_{i}\left(k;\sigma \right), \end{array}$$

(28) $$\begin{array}{cc} \; & D={\left[\begin{array}{ccc} -1 & \cdots & -1\\ 0 & \ddots & 0\\ 1 & \cdots & 1 \end{array}\right]}_{3\times m}. \end{array}$$

The vector of the pixel indices of the critical points, $I_{c}\left(j\right)$, is calculated by extracting the maximum values in each column on $S_{D}\left(i,j\right)$ as follows:

(29) $$\begin{array}{cc} I_{c}\left(j\right) & ={\mathrm{argmax}}_{i}S_{D}\left(i,j\right). \end{array}$$

(30) $$\begin{array}{c} r_{c}\left(j\right)=r_{min}+\left(r_{max}-r_{min}\right)\frac{I_{c}\left(j\right)}{n}. \end{array}$$

The critical points mean the closest point from the FLS to the object. We can replace *r* with $r_{c}$ in $p(r|\xi ,z_{P},x,m^{\prime })$, which results in

(31) $$\begin{array}{c} p\left(r|\xi ,z_{P},x,m^{\prime }\right)∼p\left(r_{c}|x,m^{\prime }\right). \end{array}$$

Then, Equation (20) is modified:

(32) $$\begin{array}{cc} w_{t}^{\left(i\right)}= & \int p\left(r_{c}|x,m^{\prime }\right)p\left(m^{\prime }|z_{P},x\right)dm^{\prime }, \end{array}$$

where (i) denotes each particle that is generated by sampling according to Equation (9).

## 3. Validation

### 3.1. Simulation

We implemented the proposed method and verified it in a simulation environment. In the simulation, we adopted a ray-tracing-based FLS simulator [17,18] and added a PS model based on the FLS model. The PS simulator emulated the mechanisms using the ray-tracing method, which was adopted to emulate the imaging mechanisms of a PS. The acoustic signals of the PS were modeled as a set of rays, surfaces of objects were modeled as a set of polygons, and reflections of acoustic signals from the surfaces of objects were considered as collisions between those rays and polygons. The distance between the PS and the point of collision $\vec{p}$ can be calculated as follows [18]:

(33) $$\begin{array}{c} {\vec{p}}_{m,n,t}=\frac{\vec{N}\cdot {\vec{p}}_{0}}{\vec{N}\cdot m,\vec{n},t}{\vec{v}}_{m,n,t}, \end{array}$$

where $\vec{N}$ is the normal vector of the collided polygon, ${\vec{p}}_{0}$ is the position vector at any point on the collided polygon, *m* and *n* are ray indices, *t* is the time index when the PS transmits an acoustic signal, and ${\vec{v}}_{m,n}$ is the direction vector of the ray with respect to *m* and *n*, which is as follows:

(34) $$\begin{array}{c} {\vec{v}}_{m,n,t}=R_{ps}R_{z}\left(\xi \right)R_{y}\left(\phi_{n}\right)R_{z}\left(\theta_{m}\right){\vec{f}}_{head}, \end{array}$$

where $R_{y}$ and $R_{z}$ are the rotation matrices with respect to the *Y* and *Z* axes, respectively, and ξ, $\phi_{n}$, and $\theta_{m}$ are the rotation angle of the PS and the azimuth and elevation angles of the ray, respectively.

(35) $$\begin{array}{cc} R_{PS} & =R_{z}\left(\gamma_{PS}\right)R_{y}\left(\beta_{PS}\right)R_{x}\left(\alpha_{PS}\right), \end{array}$$

where $\alpha_{PS}$, $\beta_{PS}$, and $\gamma_{PS}$ are the orientation angles of the PS about the X, Y, and Z axes, respectively. The intensity of the reflected ray is calculated as follows [14]:

(36) $$\begin{array}{c} I\left({\vec{p}}_{m,n,t}\right)=k\frac{z-z_{o}}{z+z_{0}}\frac{I_{0}}{\left|\right|{\vec{p}}_{m,n,t}{\left|\right|}^{2}}\cos^{2}\alpha \end{array}$$

where *k* is a unit conversion constant; *z* and $z_{0}$ are the acoustic impedances of the collision surface and water, respectively; $I_{0}$ is the reference intensity of the acoustic signals 1 m away from the profiling sonar; α is the incidence angle of the ray toward the collided polygon.

The FLS model had 96 transducers in a linear arrangement, which emitted a fan-shaped beam with 0.3${}^{\circ }$ and 14${}^{\circ }$ in the horizontal and vertical directions, respectively. The scan range of the FLS was set to 5 m, and 512 × 96 acoustic images were generated at 10 frames per second. The window start and length were set to 0.42 and 5 m, respectively. The PS consisted of a single transducer that rotated at a preset angle using a mechanical device. In addition, the PS model emitted a fan-shaped beam of 1.8${}^{\circ }$ in the horizontal and 20${}^{\circ }$ in the vertical direction. The scan speed was 3.6 ${}^{\circ }$/s. The range of the PS was 10 m, and the gain was 30 dB. To obtain the vertical profile, the PS was laid down and mounted on the upper part of the FLS. The tilt angle of both sonars was 40${}^{\circ }$ with respect to the surface.

**Figure 8:**
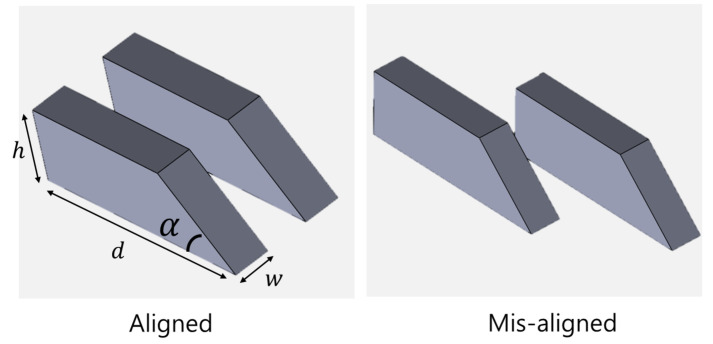
Two different deployments of objects: aligned and misaligned.

**Figure 9:**
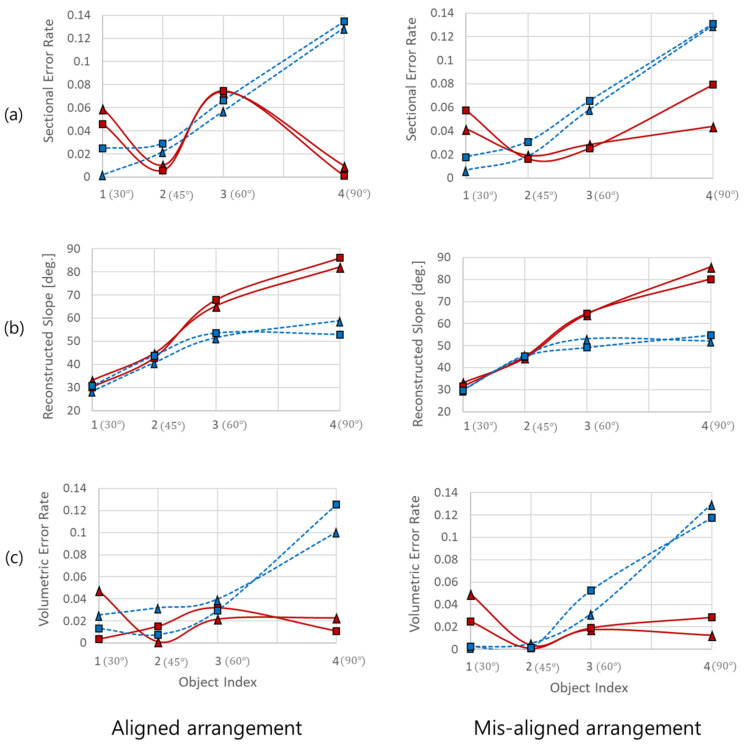
Comparison results: (**a**) sectional error rate, (**b**) reconstructed slope, and (**c**) volumetric error rate. Red and blue lines represent the proposed and single-sonar methods, respectively; solid and dashed lines represent the aligned and misaligned deployments, respectively; square and triangle symbols represent the first and second objects, respectively.

Four different objects were deployed (Table 2) with two different deployments (Figure 8). The objects had different front slopes, and they were placed side by side. This is because an FLS with vertical ambiguity experiences the reconstruction of the front area of the object. Conversely, a PS with ambiguity in the horizontal information makes it difficult to distinguish and restore two parallel objects. To evaluate the performance, we compared the cross-sectional area and front slope of the reconstructed results (Figure 9).

### 3.2. Simulation Results

**Figure 10:**
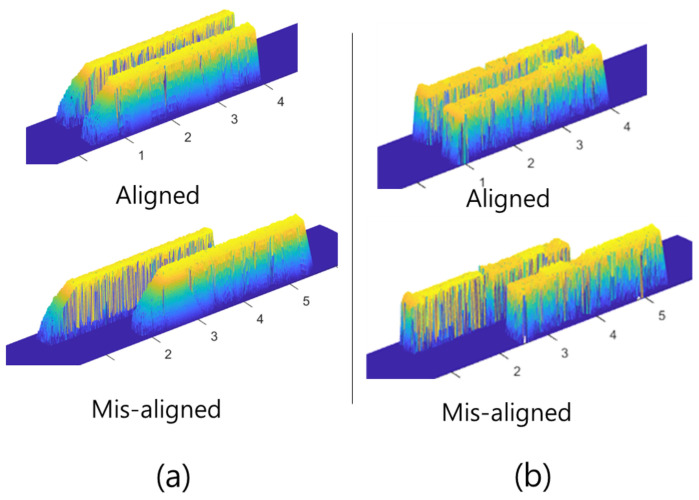
Comparison of 3D-reconstructed results for object 4: (**a**) before combination with only the FLS used and the (**b**) proposed method.

Figure 10 shows the results of the comparison of the proposed method and the single-sonar method presented in [11]. (a), (b), and (c) in each figure present the sectional area error rate, reconstructed slope, and volumetric error rate of the pointcloud reconstructed by each method, respectively. The solid red lines in each figure denote the results from the proposed method, and the dashed blue lines denote the results from the single-sonar method. Square and triangular symbols represent the first and second objects, which were placed in front and behind, respectively.

Considering the characteristics of the two sonar devices, there could be a difference in the cross-sections of the reconstructed results because the horizontal accuracy of the proposed method depends on the accuracy of the FLS, and the vertical accuracy is combined with the PS data, which could result in some differences in the horizontal accuracy of the reconstructed results. Figure 10a shows that the error of the single-sonar method increases with the slope of the object. Conversely, the error of the proposed method is bounded and does not diverge over 60${}^{\circ }$. In Figure 10b, the difference in the reconstructed slopes of the single-sonar method is significantly increased because of the ambiguity due to the vertical width of the acoustic waves, whereas it is bounded in the results of the proposed method. This tendency is valid in the comparison of volumetric error rates. We observed that it is bounded by the volumetric error rate of the reconstructed pointcloud of the proposed method.

### 3.3. Experiment

**Figure 11:**
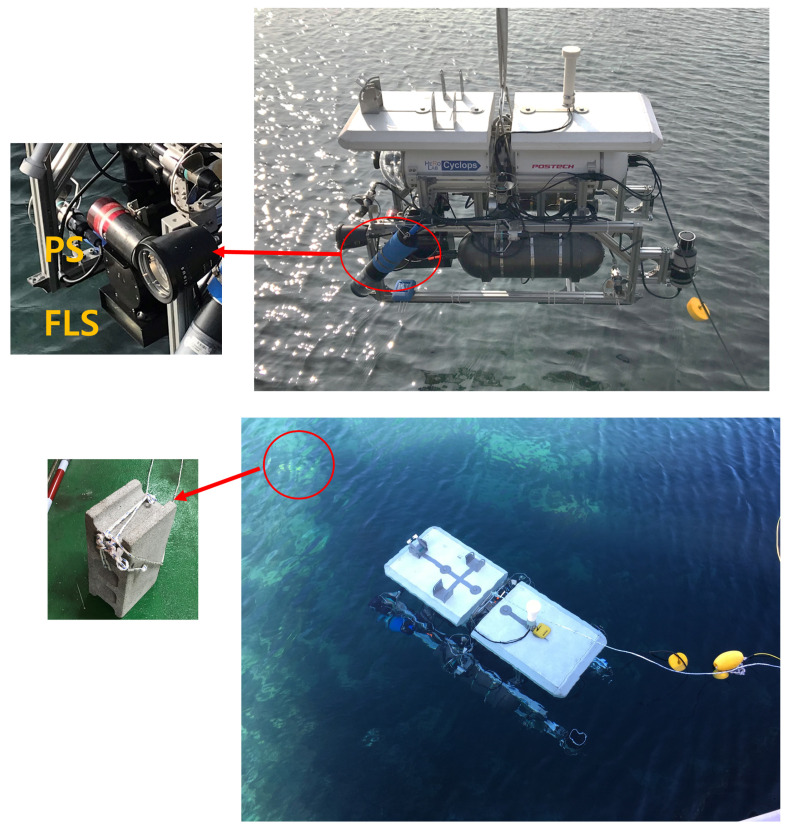
Hovering-type AUV, *Cyclops*, and the experimental setup in the field test.

We applied the proposed method to experiments using an AUV named *Cyclops* (Figure 11) developed at the Pohang University of Science and Technology (POSTECH) [19]. The *Cyclops* is a hovering-type AUV comprising eight thrusters: two for surge, four for sway, and two for heave motions. The hardware architecture of the *Cyclops* consists of two computers connected via a switching hub and sensor devices. The X-Y positions of the AUV were obtained using the doppler velocity log (DVL) [20], and the Z position was acquired using a pressure meter. The velocity accuracy of the DVL was ±0.2% ± 0.1 cm/s, and the maximum position error was ±12 cm in a minute operation. The angular orientation was measured with a fiber-optic gyroscope. The sensor system comprised an FLS, called DIDSON, a PS, a laser, and optical cameras. The sensor data were synchronized and merged with position data in the predetermined period. The environmental perception of the vehicle was mainly based on sonar devices. The control system of the vehicle had a hierarchical structure. A high-level controller supervised a low-level controller according to a mission plan, and the low-level controller followed the instructions of the high-level controller. The dynamic control system in the low-level controller was presented in [21]. The sensor data acquisition was separated into navigation sensor data and sonar data because the sonar data required a heavy computational load. For emergency situations, an emergency controller would monitor the stability of the vehicle system, and human intervention could take place by using an acoustic modem.

**Figure 12:**
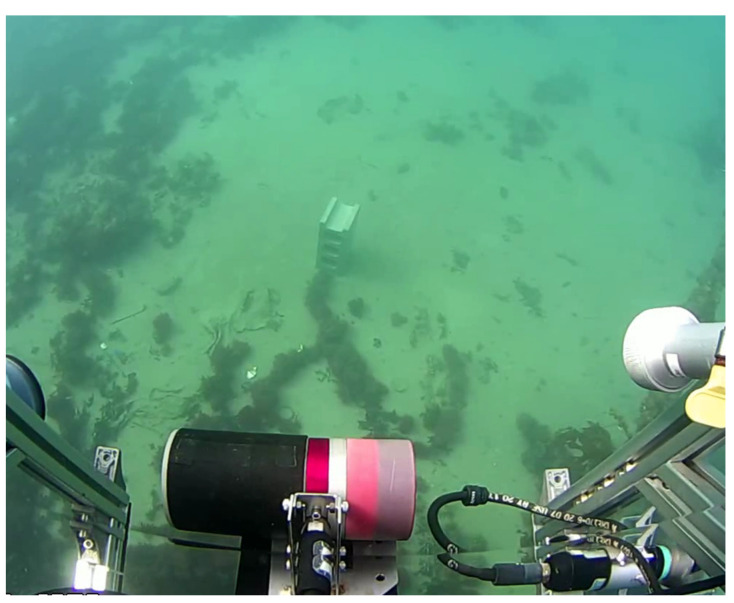
The target object captured by the camera on the AUV: concrete block (0.19 m × 0.39 m × 0.15 m (W × H × D)) deployed on the seabed.

The FLS and PS on the AUV were installed according to the specialized configuration shown in Figure 11. The PS was configured to have a maximum range of 10 m, a gain of 25 dB, a scanning sector of 60${}^{\circ }$, and a rotating speed of 1.2${}^{\circ }$/s. The FLS was set to have a window start of 0.83 m and a scan range of 5 m, and both sonars were tilted by 25${}^{\circ }$. The other acoustic specifications of the sonars were the same as those presented in the simulation section. The deployed object was a concrete brick with the size of 0.19 m × 0.39 m × 0.15 m (W × H × D). The brick was deployed on the seabed, as shown in Figure 12. The AUV with two sonar devices scanned along linear trajectories over the object at a constant altitude of 1.8 m. Sonar and AUV data were associated at a frequency of 10 Hz.

**Figure 13:**
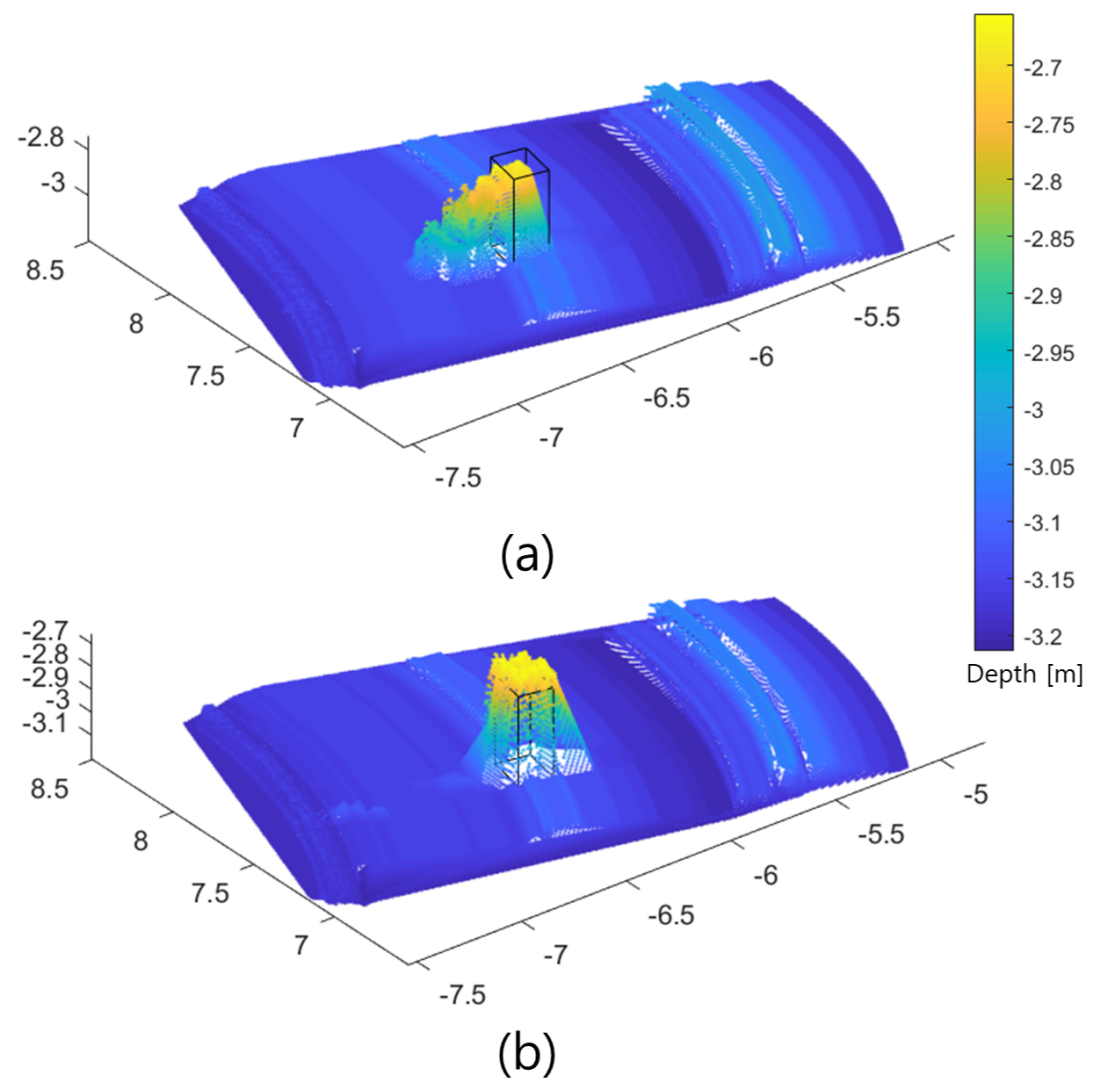
Field test results: (**a**) before combining the two types of sonar data; (**b**) the proposed method. The black solid line shows the size of the reference object.

The results are shown in Figure 13. The direction of the scan shown in Figure 12 was from left to right. Before combining the two sonar types of data (FLS only), the ambiguity of the acoustic beamwidth caused an undesired slope in front of the points, which increased the error of the 3D reconstruction (Figure 13a), whereas the proposed method improved the accuracy of the front slope and mitigated the error in the 3D reconstruction of the objects. The estimated slope of the object using the single-sonar method was 63.89${}^{\circ }$ with an error rate of 0.29, whereas that using the proposed method was 85.77${}^{\circ }$ with an error rate of 0.047. The error rates for the area of the cross-section of the reconstructed results were 0.57 and 0.12 for the single-sonar and proposed methods, respectively. Regarding volumetric errors, the error rates using the single-sonar and proposed methods were 0.81 and 0.18, respectively.

## 4. Conclusions

Herein, we presented a probabilistic sensor fusion method using two sonar devices to reconstruct elevation information from a sonar image. The FLS provides short-range scanning with a high horizontal resolution and the PS provides middle-range scanning with a low horizontal resolution, but high reliability in vertical scanning. To combine the complementary information from the two sonar devices, we presented a proposed method and conducted verification tests in a simulation and in a real sea. The field test was conducted using a hovering-type AUV equipped with sonar devices. To verify the proposed method, we compared the resulting pointcloud from the conventional method in [11] with that from the proposed method and evaluated the errors in the cross-sectional area and volume. The error rate for the cross-sectional area was improved from 0.57 to 0.12, and the volumetric error rate was also decreased from 0.81 to 0.18. The results indicate that the proposed method improved the accuracy of the generated pointcloud. This method can be utilized for pointcloud-based mapping, classification, and segmentation tasks.

## Figures and Tables

**Table 1:** Four objects used in the simulation.

Object	Front Slope [Degrees]	Dimensions (W × H × D) [m]
1	90	0.5 × 1 × 1.6
2	80	0.5 × 1 × 1.6
3	60	0.5 × 1 × 1.6
4	45	0.5 × 1 × 2
5	30	0.5 × 1 × 3
6	20	0.5 × 1 × 3

**Table 2:** Dimensions of the objects used in the simulation.

Object Index	Front Slope ${[}^{\circ }]$	Size (W × H × D, [m])	Sectional Area [m${}^{2}$]	Volume [m${}^{3}$]
1	30	0.5 × 1 × 3	2.13	1.07
2	45	0.5 × 1 × 3	2.5	1.25
3	60	0.5 × 1 × 3	2.71	1.36
4	90	0.5 × 1 × 3	3	1.5

## Data Availability

Not applicable.
